# Phenolics and Plant Allelopathy

**DOI:** 10.3390/molecules15128933

**Published:** 2010-12-07

**Authors:** Zhao-Hui Li, Qiang Wang, Xiao Ruan, Cun-De Pan, De-An Jiang

**Affiliations:** 1 College of Life Sciences, Zhejiang University, Hangzhou 310058, China; 2 Ningbo Institute of Technology, Zhejiang University, Ningbo 315100, China; 3 College of Forest, Xinjiang Agricultural University, Urumqi 830052, China

**Keywords:** phenolic compounds, allelopathy, analysis methods, mechanisms

## Abstract

Phenolic compounds arise from the shikimic and acetic acid (polyketide) metabolic pathways in plants. They are but one category of the many secondary metabolites implicated in plant allelopathy. Phenolic allelochemicals have been observed in both natural and managed ecosystems, where they cause a number of ecological and economic problems, such as declines in crop yield due to soil sickness, regeneration failure of natural forests, and replanting problems in orchards. Phenolic allelochemical structures and modes of action are diverse and may offer potential lead compounds for the development of future herbicides or pesticides. This article reviews allelopathic effects, analysis methods, and allelopathic mechanisms underlying the activity of plant phenolic compounds. Additionally, the currently debated topic in plant allelopathy of whether catechin and 8-hydroxyquinoline play an important role in *Centaurea maculata* and *Centaurea diffusa* invasion success is discussed. Overall, the main purpose of this review is to highlight the allelopacthic potential of phenolic compounds to provide us with methods to solve various ecology problems, especially in regard to the sustainable development of agriculture, forestry, nature resources and environment conservation.

## 1. Introduction

Allelopathy is a phenomenon involving either direct or indirect and either beneficial or adverse effects of a plant (including microorganisms) on another plant through the release of chemicals in the environment [1]. For over 2,000 years, allelopathy has been reported in the literature with respect to plant interference [2]. The earliest recorded observations of weed and crop allelopathy were made by none other than Theophrastus, "the father of botany", who in 300 B.C. wrote in his botanical works about how chickpea "exhausted" the soil and destroyed weeds. Cato the Elder (234–140 B.C.), the famous Roman politician and writer, was a farmer in is youth. In his book, he wrote about how chick pea and barley "scorch up" corn land. He also mentioned that walnut trees were toxic to other plants [3]. Although this form of plant–plant interference had been known for quite some time, it was only recently (1937) that the Austrian plant physiologist, Hans Molisch, gave it a formal name, allelopathy [4], and as a consequence, he is currently recognized as the father of allelopathy.

Traditionally, secondary metabolites in plants have been investigated by phytochemists. Originally classified as waste products, these compounds have recently been investigated extensively by ecologists and pharmacologists, and many complex biological functions have been discovered. Various secondary metabolites produced by plants and micro-organisms have been considered as potential allelochemicals and to play an important role in shaping interactions and communities. For example, in agroecosystems, allelochemicals have detrimental effects on the growth of associated and next-season crops [1]. In addition, weeds can exhibit allelopathy against crop plants [5]. In forest ecosystems, allelochemicals produced by invasive plants can inhibit the growth of competing vegetation through direct or indirect means, thereby providing the invader with a competitive advantage [6,7]. In addition to effects on other plants, the allelochemicals produced by invasive plants can also contribute to pest and disease resistance, and subsequently confer a competitive advantage to the invader in the host range [8]. 

**Figure 1:**
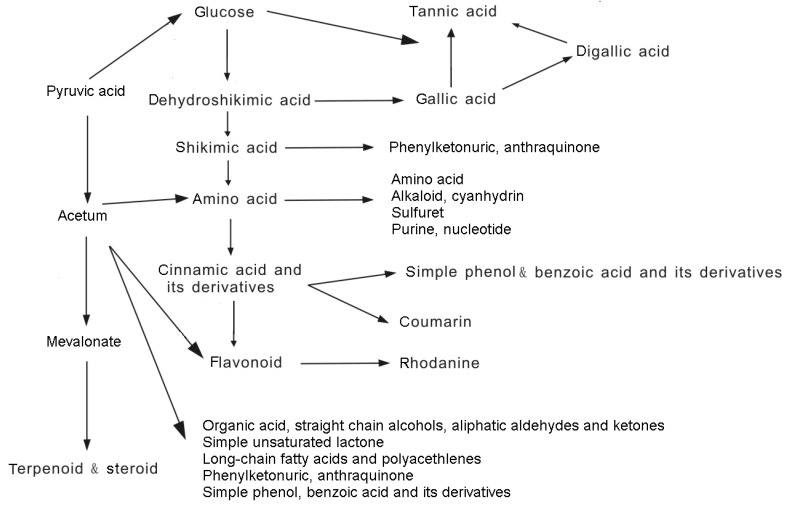
Biosynthetic pathways of major allelopathic substances [9].

According to the different structures and properties of these compounds, allelochemicals can be classified into the following categories: (1) water-soluble organic acids, straight-chain alcohols, aliphatic aldehydes, and ketones; (2) simple unsaturated lactones; (3) long-chain fatty acids and polyacetylenes; (4) quinines (benzoquinone, anthraquinone and complex quinines); (5) phenolics; (6) cinnamic acid and its derivatives; (7) coumarins; (8) flavonoids; (9) tannins; (10) steroids and terpenoids (sesquiterpene lactones, diterpenes, and triterpenoids). The biosynthetic pathways of the major allelopathic substances are shown in Figure 1 [9]. 

Phenolic compounds are a class of the most important and common plant allelochemicals in the ecosystem. They are chemical compounds consisting of a hydroxyl group (-OH) bonded directly to an aromatic hydrocarbon group. Within the context of allelopathy, the term “phenolic compounds” has a loose meaning, but it is generally thought of as containing a range of compound types that include structures such as simple aromatic phenols, hydroxy and substituted benzoic acids and aldehydes, hydroxy and substituted cinnamic acids, coumarins, tannins, and perhaps a few of the flavonoids [3]. They have been the subject of a great number of chemical, biological, agricultural, and medical studies. Recent interest in phenolic compounds stems from their potential protective role (*i.e*., through the ingestion of fruits and vegetables), against oxidative damage caused diseases, such as coronary heart disease, stroke, and cancers. However, mach evidence has show that phenolic compounds are involved in plant allelopathy. They are universally distributed in plants and very common in plant decomposition products, and they are important precursors of humic substances in soils. In soil, phenolics can occur in the three following forms: free, reversibly bound, and bound forms. *ortho*-Substituted phenolics, such as salicylic and *o*-coumaric acids, and dihydro-substituted phenolics, such as protocatechuic and caffeic acids, are adsorbed by clay minerals by forming chelate complexes with metals. Free phenolic compounds may accumulate in rhizosphere soils, especially in soils flooded with vegetable waste waters, thereby influencing the accumulation and availability of soil nutrients and rates of nutrient cycling, which both ultimately affect plant growth. Studies have reviewed the chemistry, biotechnology, and ecotoxicology of naturally occurring polyphenols in vegetable waste [10]. One of the best known examples of the protective role of phenolics was that of protocatechuic acid and cathecol from onion, which were known to aid against infection of *Colletotrichum circinaus* [11]. Water soluble phenolics diffused out from the dead cell layers of the scales and inhibited spore germination and/or hyphal penetration of the pathogen. 

## 2. Origin and Allelopathy Effects of Phenolics

Structural diversity and intraspecific variability represent the most significant characteristics of phenolic compounds [12]. Biosynthesis and accumulation of phenolic compounds arise from highly regulated processes, which require cell-, tissue-, development-, and environment-specific controls. The pathways for phenolic compounds have been selected over time among specific plant lineages, especially when these compounds take over specific advantageous functions. These functions include scent and colour to attract pollinators or to ward off herbivores or pathogens [13]. Phenolic compounds generally arise from the pentose-phosphate pathway. 4-Phosphate erythrose and phosphoenolpyruvic acid undergo condensation reactions with 7-phosphate altoheptulose, which generate phenolic compounds after a series of transformation steps in the shikimic and acetic acid (polyketide) metabolic pathways.

Phenolic compounds are used in several industrial processes to manufacture chemicals such as pesticides, explosives, drugs and dyes. They are also used in the bleaching process of paper manufacturing. Apart from these functions, phenolic compounds have substantial allelopathic applications in agriculture and forestry as herbicides, insecticides, and fungicides [14]. 

**Figure 2:**
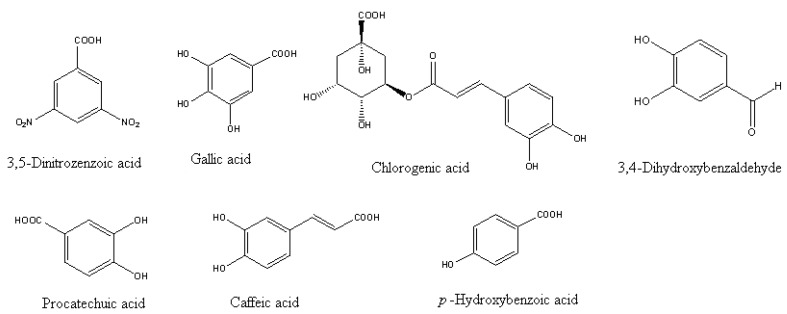
Main phenolic allelochemicals isolated from aqueous extracts of *D. regia* [15].

*Delonix regia* has been planted in many places in the south of Taiwan as an ornamental tree. Its allelopathic potential has been bioassayed and the 1%, 2%, 3%, 4%, and 5% aqueous extracts of flowers, leaves, and twigs of donor plants caused the growth of lettuce (*Lactuca sativa*) and Chinese cabbage (*Brassica chinensis*) to be significantly inhibited compared with distilled water treated controls [15]. Subtracting 20% osmotic and pH inhibition, the inhibitory effects of these aqueous extracts from donor plant parts were still significantly more than 30 %. These results demonstrated that *D. regia* showed strong allelopathic potential and the effects of osmolality and pH could be neglected. The following allelochemicals were the principal compounds isolated from the aqueous extracts: chlorogenic acid, protocatechuic acid (3,4-dihydroxybenzoic acid), gallic acid, 3,4-dihydroxy- benzaldehyde, *p*-hydroxybenzoic acid, caffeic acid (3,4-dihydroxycinnamic acid) and 3,5-dinitrobenzoic acid (Figure 2). 

To further confirm the allelopathic properties of these isolated phenolic compounds, the following compounds were bioassayed with lettuce seeds: chlorogenic, gallic, *p*-hydroxybenzoic, protocatechuic, caffeic, 3,5-dinitrozenzoic acids and 3,4-dihydroxybenzaldehyde (all prepared in a series of concentrations of aqueous solutions at 10, 20, 40, 80, 160, 320, and 500 ppm). Bioassay results indicated that there was significant inhibition above 30% of the control at 10 ppm. The inhibition increased when the concentration of compounds was increased [15]. Most phenolic compounds have been confirmed as allelochemicals in previous reports [16,17,18,19,20]. Another bioassay of the aqueous extract of *D. regia* rhizosphere soil indicated that the main phenolic compounds were water soluble and leached out from the litter on the soil surface and were carried down to the deep soil by rainfall [15].

**Figure 3:**
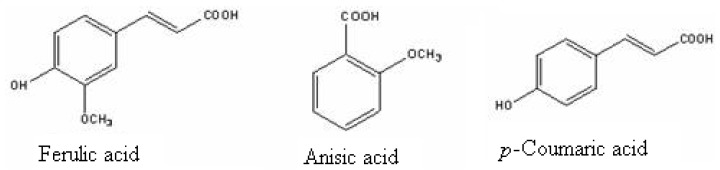
Three phenolic allelochemicals isolated from *A. conyzoides* rhizosphere soil [21].

The main allelochemicals in the rhizosphere soil of *Ageratum conyzoides* L. (billy goat weed; *Asteraceae*) were isolated and identified to be *p*-coumaric acid, gallic acid, ferulic acid, *p*-hydroxybenzoic acid, and anisic acid (Figure 3) [21]; the concentrations of these phenolic compounds in the rhizosphere soil free from plant debris were 24.2 ± 3.08, 17.8 ± 0.19, 36.3 ± 0.53, 21.5 ± 0.34, and <0.1 ug/g soil (trace amount), respectively. However, the actual amounts in the soil may be higher than those determined. Five seeds from a rice test plant (*Oryza sativa* L. var. no. 3) were cultured in the rhizosphere soil of *A. conyzoides* (treatment) with a control (riverbed sand without any previous growth of *A. conyzoides*) in an environmentally controlled growth chamber. Differences in growth could be seen after one month. Growth of rice measured in terms of root and shoot (coleoptile) length and seedling weight was significantly reduced in the rhizosphere soil. The root length, shoot (coleoptile) length, and seedling dry weight of these rice plants were inhibited by 30.2, 21.7 and 16.3% compared with control, respectively. These findings indicated that root residues of *A. conyzoides* released inhibitory substances in the rhizosphere soil and these phytotoxins negatively affected rice growth. The quantitative analyses of putative phytotoxins in the rhizosphere soil showed it contained nearly six times more phenolics than the control soil. However, the amount of phenolics in the rhizosphere soils declined signiﬁcantly when activated charcoal was added. Activated charcoal has a great afﬁnity for phenolic metabolites and does not adsorb inorganic molecules [22].

**Figure 4:**
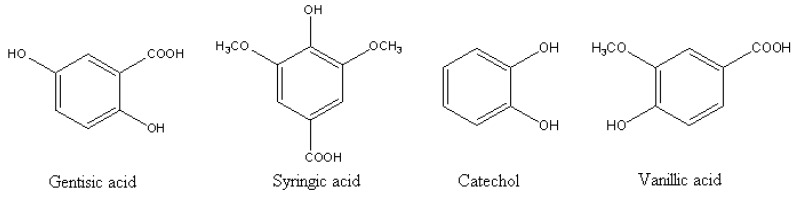
Four phenolic allelochemicals isolated from Eucalyptus [23].

Eucalyptus belongs to the family Myraceae, mostly found in tropical region, and is a native to Australia. The tree is considered to have allelochemicals and volatile compounds in its all parts. These chemicals have harmful effects on the crops in the ecosystem resulting in the reduction and delaying of germination, mortality of seedling and reduction in growth and yield [22]. Investigations done to identify the allelopathic compounds in the leachates of bark, fresh leaves and leaf litter of *Eucalyptus tereticornis*, *E. camaldulensis*, *E. polycarpa* and *E. microtheca* showed the presence of *p*-coumaric, gallic, gentisic, *p*-hydroxybenzoic, syringic and vanillic acids and catechol (Figure 4) [23]. 

Bioassay results of the identified phenolics on blackgram (*Phaseolus mungo L.*) showed that seed germination of blackgram was affected by catechol both at 1 mM and 2 mM concentrations. Gallic acid at 1 mM concentration and syringic and hydroxybenzoic acids at 2 mM concentrations significantly inhibited the germination. All the phenolic acids except coumaric acid, significantly decreased the vigour index both at 1 mM and 2 mM concentrations. 

## 3. Analysis Methods for Phenolic Allelochemicals

Most of the phenolics identified as allelochemicals have been extracted from plant materials. Early in the 19th century, de Candolle (1830) observed cropping obstacles and the potentiating or inhibition effects of root exudates on the neighboring plants [25]. However, due to the limited analytical methods of the time, it was difficult to identify the allelochemicals. Not until the 1980s were various methods developed for the detection and quantification of phenolic compounds, which included such techniques as planar chromatography, column chromatography on silica, modified silica or dextran gels, ion exchange chromatography, capillary electrophoresis, and counter-current chromatography [26]. Initially, there were various trace phenolic compounds identified as allelochemical. It was necessary to collect, extract, separate and then analyze phenolics in the natural environment to bioassay their phototoxicity.

### 3.1. Extration Methods of Total Phenolics with Solvents

Organic solvents, acids and bases have always been used for the extraction of phenolics from plant materials with different efficiencies for individual classes of phenolic compounds [27]. Polar solvents such as methanol, ethanol, acetone, or acetonitrile give much high extraction efficiencies, but they also extract other undesirable polar compounds present in the samples. With a polar solvent, such as chloroform or dichloromethane, the extraction of phenols requires a previous acidic digestion of the analytes. For extraction, the Soxhlet extraction has been one of the most popular techniques for isolating phenolic compounds from solid samples. This was probably due to its simplicity and the inexpensive extraction apparatus. Ultrasonic extraction has been shown to be another conventional technique to extract analytes from solid samples. Although sonication has been shown to be faster than Soxhlet extraction, it also requires large volumes of toxic and expensive organic solvents. However, such methods do not provide correct data either on the amount of phenolics compounds or on their qualitative composition. Although most plant tissues contain potential allelochemicals, only those compounds released from the plant into the environment are available to exert an allelopathic effect on another organism [28]. Therefore, water is the only extracting solvent present in Nature and standing at room temperature with water should be preferred over any other extraction method for the extraction of phenolics [29]. Some studies found that there was no significant correlation between the level of allelochemicals detected within wheat roots and the level in wheat root exudates [30].

However, there still remains the problem of relating plant extracts to allelopathic effects. Certain compounds present in plant components may show an inhibitory effect on test species but may not leach or exude from the plant under natural conditions. To establish the actual involvement of phenolics in allelopathy, it was desirable to collect the following data: (1) bioactive concentration of phenolics in the medium (*i.e*., whether the concentrations at which they were active is actually present in the environment), (2) the residence time and the static and dynamic availability of the phenolics, and (3) the additive or partially antagonistic activity of the phenolic compound [31].

### 3.2. Separation and Detection Methods of Total Phenolics

#### 3.2.1. Liquid-liquid Extraction

The current common analytical methods used to extract phenolic compounds from liquid samples were based on liquid-liquid extraction (LLE) followed by gas chromatography (GC) determination with different detection methods. This technique offers efficient and precise results; however, it is relatively time-consuming, possibly harmful due the use of large volumes of organic solvents (frequently toxic), and highly expensive [14].

#### 3.2.2. Chromatography

In 1910, the Russian botanist Mikhail Tsvet was the first to describe column chromatography, which was essentially performed then in the same way it is practiced today. Specifically, he filled a vertical glass column with an absorptive material, such as silica, alumina, or powdered sugar, added a solution of the compound mixture to be purified on top of it (plant pigments) and washed them through the column with organic solvents. The pigments separated into a series of discrete bands on the column that were divided by free regions. Tsvet worked with colored plant pigments and coined the terms carotenoids and chlorophyll. That was why he called the method chromatography. Today, ecologists interested in obtaining pure phenolic allelochemicals from complex mixtures can choose from diverse technologies, which include HPLC, GC, LC-MS, GC-MS, TLC, CE, paper chromatography, among others.

HPLC has been successfully employed in quantitative and qualitative analyses of phenolics. The small particle sizes of column material (*i.e*., 5 µm and below) afford near-baseline separations of the majority of analytes. Computerised spectroscopic detectors, which allow the continuous acquisition of on-line spectra during a chromatographic run with particular diode-array UV technology, have further revolutionised this methodology. Thus, this technique has become the ultimate choice of analysis of nonvolatile phenolics such as flavonoids and coumarins [32]. GC is another powerful separation technology available today but is limited to volatile and semivolatile compounds [13]. However, this method has led to a technical breakthrough when combined with the application of mass spectrometry as a chromatography detector. GC-MS analysis can identify pure compounds present at less than 1 ng [33]. GC-MS-MS is superior to GC-MS for the analysis of complex mixtures owing to its enhanced selectivity and sensitivity [34]. MS-MS is preferential for low molecular weight molecules (<1000 amu) in complex mixtures due to its high specificity for target analytes and decreased susceptibility to mass interferences [35]. Notably, the power of the GC-MS-MS techniques to analyze complex mixtures, such as those found in rice root exudates, has been gaining attention [36].

## 4. Allelopathy Mechanisms of Phenolics

### 4.1. Changes in Membrane Permeability and Inhibition of Plant Nutrient Uptake

**Figure 5:**
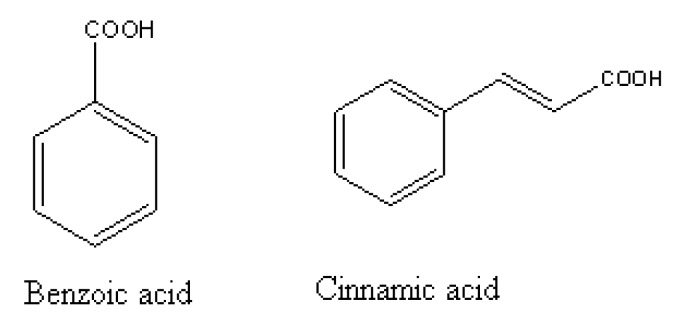
Two phenolic allelochemicals for cucumber bioassays [37].

Phenolic allelochemicals can also lead to increased cell membrane permeability. Consequently, cell contents spill and there is increased lipid peroxidation. Finally, there is slow growth or death of plant tissue. In addition, phenolic allelochemicals can also inhibit plants from absorbing nutrients from surroundings and affect the normal growth of plants. A study treated cucumber (*Cucumis sativus*) with benzoic acid and cinnamic acid derivatives (Figure 5) for seven days [37]. The results showed that declines in phenols glycosylation and phenyl-ß-glucosyltransferase (PGT) activity decrease were linked with increases in membrane permeability. 

### 4.2. Inhibition of Cell Division, Elongation, and Submicroscopic Structure

**Figure 6:**
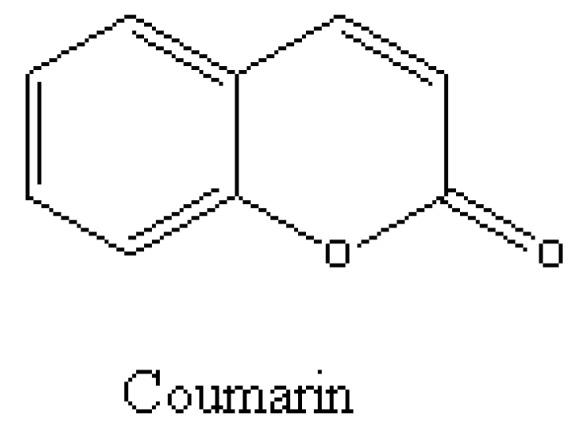
Coumarin for lettuce bioassays [39].

It was found that phenolic allelochemicals could inhibit plant root elongation, cell division, change cell ultra-structure, and then interfere with the normal growth and development of the whole plant. In a study of allelopathic effects on *Phaseolous vulgaris*, Cruz *et al.* [38] found that *P. vulgaris* root tip cells were extruded together and that cell organization was disordered with little cell differentiation. Li *et al.* [39] found that coumarin significantly inhibited the root elongation of lettuce (*Lactuca sativa* L.) seedlings (Figure 6), significantly increased the thickness of the cortex cells, and reduced cellular activity and the amount of Golgi body. After a 7-day treatment with benzoic acid, Eutrema wasabi root elongation was inhibited up to 81.1%. The root cells were irregularly arranged and organelle structures were severely damaged.

### 4.3. Effects on Plant Photosynthesis and Respiration

The impact of phenolic allelochemicals on the respiration of plants has mainly been shown to involve weakened oxygen absorption capacity, while the impact on photosynthesis has mainly been to reduce the chlorophyll content and photosynthetic rate. Patterson [40] reported that 10–30 µmol/L caffeic acid, coumaric acid, ferulic acid, cinnamic acid, and vanillic acid could significantly inhibit the growth of soybean (*Glycine max*). Photosynthetic products and chlorophyll content of *G. max* were also strongly reduced. Another study incubated cucumber seedlings in solutions containing derivatives of benzoic and cinnamic acids [41]. The results showed that leaf transpiration, stomatal conductance, and the intercellular CO_2_ concentrations were all decreased.

### 4.4. Effects on Various Enzyme Function and Activities

**Figure 7:**
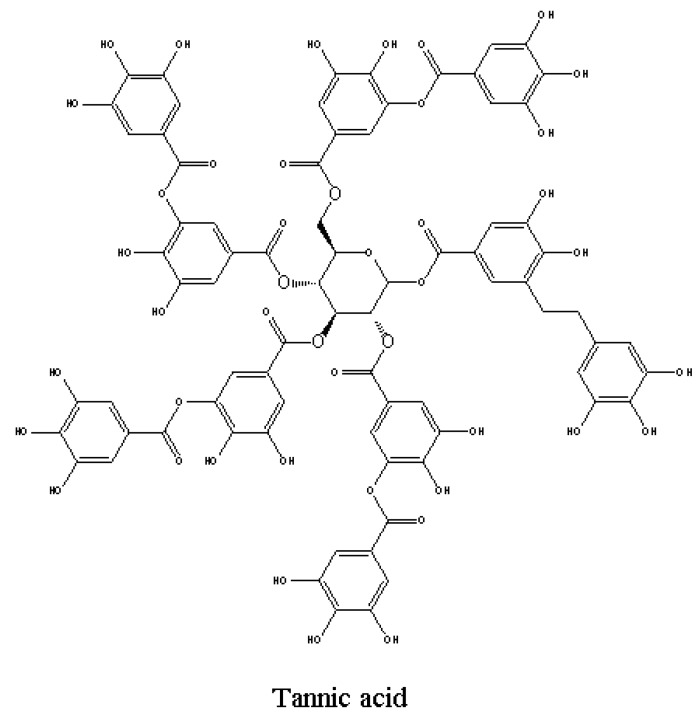
Structures of tannic acid for plant enzyme-activities bioassays [42].

Phenolic allelochemicals enter through the plant cell membrane and change the activity and function of certain enzymes. Previous results [42] have demonstrated the following enzymatic mechanisms: chlorogenic acid, caffeic acid and catechol can inhibit activities of phosphorylase; cinnamic acid and its derivatives can inhibit the hydrolysis activities of ATPase; tannic acids (Figure 7) can inhibit activities of peroxidase (POD), catalase and cellulose. Some of the recent studies reported that phenolics can affect the activities of POD and phenylalnine ammonialyase (PAL) [43]. The activity of POD increased 18% and 47% when treated with vanillic acid at 0.5 mM and 1mM, respectively. However, the activity of PAL decreased 32% when treated with vanilla acid at 1mM. Batish *et al*. reported that the activity proteases in hypocotyl cuttings of mung bean (*Phaseolus aureus*) decreased significantly at 1mM caffeic acid treatment [44]. Caffeic acid at 0.1mM also induced generation of reactive oxygen species (ROS) and resulted in a signiﬁcant change in the activities of POD, that caused reduction of rhizogenesis and suppressed root growth of mung bean hypocotyl cuttings. Results of other studies reported that root length and fresh weight of maize (*Zea mays* L.) seedlings were significantly reduced after a 6-day treatment with ferulic acid [45]. In addition, activities of hydrolase, maltase, phospholipase, and protease were also significantly reduced.

### 4.5. Effects on Synthesis of Plant Endogenous Hormones

Phenolic allelochemicals can reduce or inactivate the physiological activity of plant hormones, which may then inhibit the normal physiological process of plants. He *et al.* found that hydroxyl benzoic acid, polyphenols, and other compounds could affect the decomposition process of indoleacetic acid and gibberellin [46]. In addition, aqueous extracts of *O. sativa* can increase indoleacetic acid oxidase activity of receptor plants, and then reduce the level of indoleacetic acid [47]. Some studies also found that salicylic acid could inhibit ethylene synthese in pear (*Pyrus communis*) using a cell suspension culture study [48].

### 4.6. Effects on Protein Synthesis

**Figure 8:**
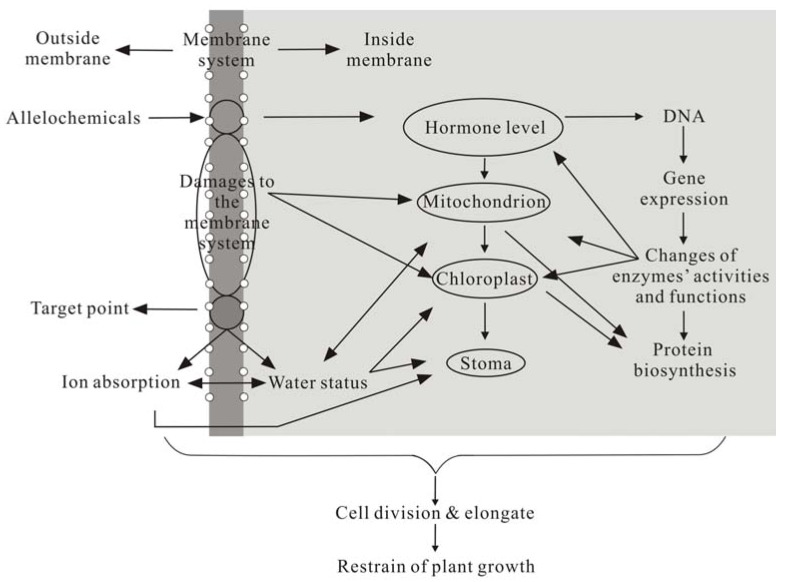
Mechanism of allelochemicals [9].

Some phenolics (*i.e*., ferulic acid and cinnamic acid) can inhibit protein synthesis [46]. Phenolic allelochemicals from *O. sativa* can inhibit amino acid transport and protein synthesis, and the subsequent growth of treated plants. All phenolics could reduce integrity of DNA and RNA [47,49]. However, in the vast majority of cases, phenolic compounds appear as a mixture and not as a single substance. Hence, the contribution made to allelopathy by phenolic compounds was probably never due to a single substance [50]. A series of physiological and biochemical changes in plants induced by phenolic compounds are shown in Figure 8 [9].

## 5. The Forefront of Plant Allelopathy and Our Work

### 5.1. Are (±)-Catechin and 8-Hydroxyquinoline Putative Allelochemicals Underlying Knapweed’s Invasion Success?

Over the past ten years, the scientific dabate of whether catechin is a putative allelochemical of *Centaurea maculosa* has attracted extensive interest. *Centaurea maculosa* Lam. (spotted knapweed, recently suggested to be *C. stoebe* L. [USDA, NRCS 2007]) is a Eurasian species of *Asteraceae* that has invaded North American grasslands. It has been shown to reduce native biodiversity and rangeland forage quality [51,52]. The notion of a possible role of allelopathy in the invasiveness of *C. maculosa* has received considerable attention. Allelopathy has been proposed to contribute to the effectiveness of some plant invasions because they may produce chemicals for which native species have not yet evolved resistance (*i.e*., the novel weapons hypothesis) [6,53]. Initial studies of *C. maculosa* allelopathy have indicated that phytotoxins in *C. maculosa* root exudates may inhibit native species [7].

**Figure 9:**
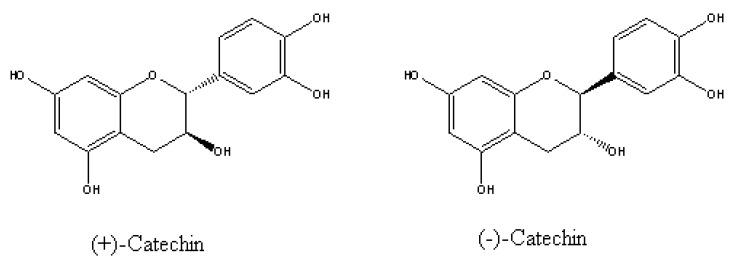
Structures of both enantiomers of catechin in *C. maculosa* root exudates [54].

Efforts to identify phytotoxins in *C. maculosa* root exudates revealed an important racemic mixture of (±)-catechin (Figure 9) [54], which was the first allelochemical isolated from knapweed by the Callaway team. Since then, several studies have reported high (±)-catechin concentrations in *C. maculosa* soils [54,55,56,57,58]. (±)-Catechin also has been reported to inhibit growth and/or reduce survival of susceptible North American plants *in vitro* [54,55,59,60], in soil in controlled environments [55], and in soil in the field [61]. (±)-Catechin at high concentrations has been reported to inhibit *C. maculosa* growth and germination, thereby acting as an autoinhibitor [56,60]. It was also reported that although spotted knapweed roots exude (±)-catechin, only the (-)-catechin enantiomer was phytotoxic. (+)-Catechin has antibacterial activity against root-infesting pathogens, which (-)-catechin does not show [54]. It has demonstrated the mechanism of inhibition on native species’ growth and germination in ﬁeld soils at natural concentrations of (–)-catechin (0.35 mM) [55]. In susceptible species such as *Arabidopsis thaliana*, the allelochemical triggered a wave of reactive oxygen species (ROS) initiated at the root meristem, which led to a Ca^2+^ signaling cascade triggering genome-wide changes in gene expression and, ultimately, death of the root system. Therefore, (-)-catechin secreted by the roots of *C. maculosa*, plays an important ecological role in interaspecific and interspecific interactions, especially in the success of invasive *C. maculosa* in North American.

Other recent studies have reported much lower soil (±)-catechin concentrations or have failed to find (±)-catechin in *C. maculosa* soil [62,63]. Furthermore, some studies have reported weak or no effects of high (±)-catechin concentrations on the same species that other studies have reported strong effects [60,61,62]. For example, Weir *et al.* [59] reported 100% mortality of Idaho fescue at 0.17 mM, while Blair *et al.* [62] only reported reduced growth of this same species at 3.1 mM. Duke *et al.* [64] found that (±)-catechin stunted the roots of *A.* *thaliana* grown continuously on agar with concentrations greater than 0.35 mM. In contrast, 3.3 mM (±)-catechin had no effect on root growth of lettuce. Tharayil *et al.* [65] reported no effect of (±)-catechin on lettuce growth up to the limits of its solubility. Some of these disparities could be due to differences in growing conditions, method of treatment, and/or biotype or variety assayed. Bais *et al.* [54] reported that (−)-catechin was 1.5- to 2-fold more active than the positive (+) enantiomer. However, in a similar experiment to that of Bais *et al.* [54], Duke *et al.* [64] found that the two enantiomers had similar activity, although (−)-catechin was slightly more active. Blair *et al.* [63] analysed (±)-catechin concentrations in soil samples taken from three long-term knapweed infested sites in Montana, USA, during the summer and fall of 2005. The results showed that (±)-catechin was only detected in two of the soil cores site at one time point (August, 2005). The field levels from these two sites were nearly three orders of magnitude lower than what had been previously reported to cause reduced growth in a sensitive native species. Fourteen percent of the remaining soil cores contained low but detectable levels (<0.35 μM) of (±)-catechin [63]. These results were in sharp contrast to recent reports of catechin found in the field at levels ranging from～4.7 to 9.5 mM on average with a single high value reported at 22 mM (also from Montana, USA) [55,56,57].

It is known that (±)-catechin is highly unstable and degrades rapidly at neutral and basic pH in both water and soil, and even in acidic soils if they are moist [66,67,68]. Thus, the level of (±)-catechin available to cause a biological effect may be much smaller than what is actually applied. The same could be said of catechin in solution. If (±)-catechin is the causal agent of any effect, its instability could contribute to its very weak effects. However, the debate as to whether (±)-catechin is a viable allelopathic agent is currently receiving increased attention, and more and more papers shed doubt on the importance of this chemical in the success of invasive spotted knapweed. 

**Figure 10:**
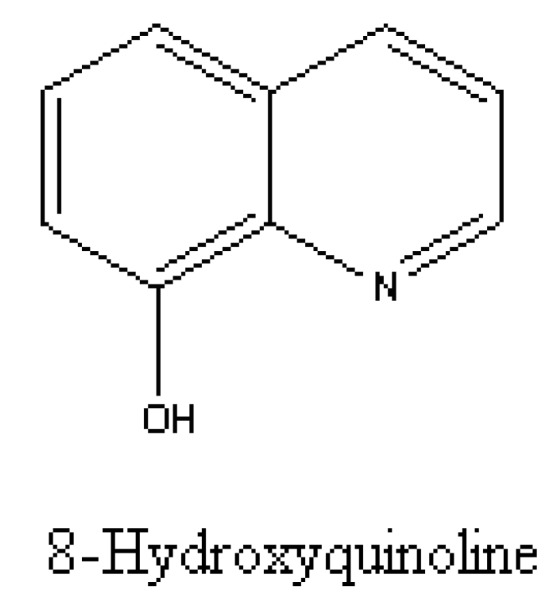
Structure of8-hydroxyquinoline isolated from *C. diffusa* root exudates.

This is not the only ongoing debate about putative allelochemical production by kapweeds. 8-Hydroxyquinoline was another phenolic allelochemical isolated from knapweed by the Callaway team (Figure 10). Vivanco *et al.* [69] found that 8-hydroxyquinoline was at least three times more concentrated in *Centaurea diffusa* Lam. that had invaded North American soils (221.61 ± 12.4～264.12 ± 21.2 μg/g soil DW) than in this weed’s native Eurasian soils. In addition, it had stronger phytotoxic effects on grass species from North American than on grass species from Eurasian. This allelochemical also displayed strong antibacterial and antifungal activity against important pathogenic microbes such as *Aspergillus niger*, *Rhizoctonia solani, Pbytopbtbora infestans*, *Fusarium oxysporum*, *Xantbomonas compestris*, *etc*. Thus, *C. diffusa* benefited from 8-hydroxyquinoline both as a phytotoxin and as an antimicrobial. In the experiment similar to Vivanco *et al.* [69], Norton *et al.* [70] detected the presence of 8-hydroxyquinoline in 12 field-collected soils but was unable to detect the compound in any of the sites. Therefore, it was concluded that 8-hydroxyquinoline was not the responsible allelochemical in *C. diffusa* invasion success, since *C. diffusa* could not produce the allelochemical at ecologically meaningful concentrations.

There are many factors that may have influenced these experimental results, which include sampling sites and time, extraction solvent, analytical methods, bioassay conditions, and so on. Overall, chemical interference is probably be based upon a combination of phytotoxic metabolites, not only one or two phytotoxins, and is influenced a wide range of metabolic pathways. Further studies on the phenomenon of knapweed’s successful invasion should be carried out in invasion fields and rigorous bioassay-directed isolation of responsible compound(s) might solve this mystery.

### 5.2. Several International Allelopathy Research Centers

Natural products, such as allelochemicals isolated from plants, offer a vast, virtually untapped reservoir of chemical compounds with many potential uses. One of these uses is in agriculture to manage pests and/or weeds with less risk than with synthetic compounds that are toxicologically and environmentally undesirable. Labs of Natural Products Utilization Research Unit of the National Center for the Development of Natural Products, led by Stephen O. Duke, are now focused on discovery of natural products for pests and/or weeds management in agriculture in order to produce more toxicologically benign pests and/or weeds management tools, and to improve the nutriceutical value of crops. They have successfully isolated potential allelochemicals and investigated their modes of action, such as 1, 8-cineole (isolated from *Eucalyptus* spp.) [71,72], *p*-coumaric acid (isolated from *O. sativa*) [73], and cyperin (isolated from plant pathogens) [72].

Dr. Ragan M. Callaway serves as leader of plant ecology lab of the University of Montana. The primary focus of the research in the lab is on interactions among plants. These include direct interactions, such as competition for resources, allelopathy, and facilitation; and indirect interactions mediated by herbivores, soil microbes, and other plants. The lab has isolated important allelochemicals, (±)-catechin and 8-hydroxyquinoline, from knapweeds, and researched their allelopathic mechanisms [55,56,57,58,69].

Yoshiharu Fujii team, from the National Institute of Agro-Enviromental Sciences in Japan, is focused on allelopathy in crop and weed management, and allelochemicals in sustainable agriculture. The team evaluated the allelopathic potential of mesquite (*Prosopis juliﬂora* (Sw.) DC.) [74], buckwheat (*Fagopyrum tataricum*) [75] and Oxalidaceae [76]. The research results showed that (–)-lariciresinol [74], catechin, gallic acid [75] and quercetin( [77] were all potential allolechemicals, and provided alternatives to develop environmental friendly pesticide and herbicide.

Inderjit, from the Centre for Environmental Management of Degraded Ecosystems in Delhi University, India, has been devoted to the study of allelopathy for twenty years. He has mainly provided some allelopathy research methods for laboratory and field bioassays [78,79,80]. Jing-quan Yu’s team, from the Department of Horticulture at Zhejiang University, P.R. China, is focused on the autotoxic potential in horticultural crops and allelapthic suppression of soil-borne diseases in cropping systems. Yu studied the autotoxicity of Cucurbitaceae, especially cucumber (*Cucumis sativus*) [81,82]. He reported that cinnamic acid could cause oxidative stress in cucumber roots, suppress photosynthesis of root cells, and promote incidence of *Fusarium* wilt [83,41].

### 5.3. Our Work on Schrenk Spruce

The Schrenk spruce (*Picea schrenkiana* Fisch. et Mey.) forest is one of the most important zonal vegetations in the Tianshan Mountains in Northwest China. It makes up 98.6% of the total stand volume in the region, and plays a critical role in soil and water conservation. It is mainly distributed at elevations between 1,600 and 2,800 m. Schrenk spruces grow slowly and often form stands with uneven distributions of size and age. In addition, regeneration of these stands has been a problem and productivity declines associated with Schrenk spruce forest have also been observed, which are probably due to resource competitions [84]. The accumulation of phytotoxic substances in the rhizosphere (*i.e*., leaf leachates and root exudates and /or metabolites) may have also contributed to the observed regeneration problems and productivity decline [85]. Our results have showed that litter extracts from spruce affected seed germination and seedling growth of the same species.

**Figure 11:**
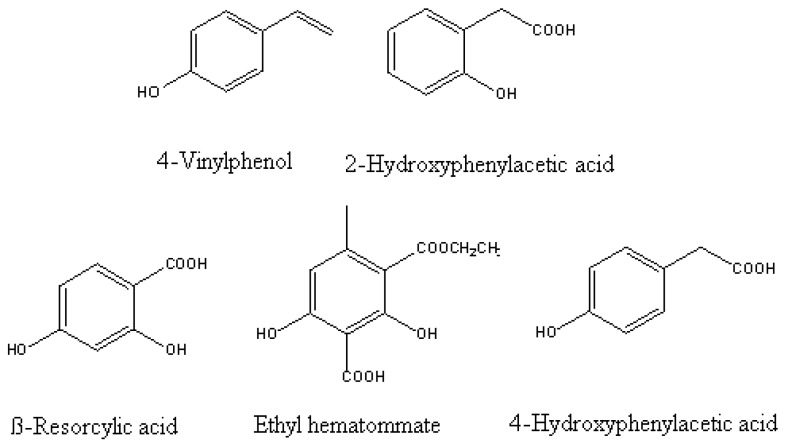
Some puative allelochemicals isolated from aqueous extract of *P. schrenkiana* litters.

The senescent leaves contained an array of allelochemicals that were inhibitory to spruce seed germination and seedling growth. These findings led us to isolate allelochemicals from these litters. In total, there were 17 components that were isolated. Phenolic acids occupied a large proportion of the potential allelochemicals that were detected in litter aqueous extracts that best correlated with the observed allelopathic effect. Of the identified potential allelochemicals, 10 were phenolic acids, which included 4-vinylphenol, *p*-hydroxybenzoic acids, 2-hydroxyphenylacetic acid, vanillic acid, gallic acid, gentisic acid, 4-hydroxyphenylacetic acid, ß-resorcylic acid, *p*-coumaric acid, and ethyl hematommate (Figure 11). 

Litter extracts were a mixture of mostly lower molecular weight substances and their metabolites (*i.e*., phenolic compounds, tannins, indoles and other compounds). According to Appel, the expression of phytotoxic effects was dependent on the threshold concentration of phytotoxins in soil [86]. Rainfall, microorganisms and plant metabolism, are all factors influencing the allelochemical concentrations in soil. Microbial metabolism is an important factor in determining the magnitude and duration of allelopathic interactions involving phenolic acids present in soils through the release of phenolic acids from organic residues or through the reduction of soil concentrations of phytotoxic phenolic acids [1,87]. Simple phenolic acids, such as ferulic and *p*-coumaric acid, can be utilized by microbial and transformed to other phenolic acids, such as vanillic acid, *p*-hydroxybenzoic acid, and/or protocatechuic acid before the aromatic ring structure is broken, and then these phenolic acids can not be allowed for accumulation to phytotoxic levels [88,89,90]. Our study only carried out controlled laboratory experiments and no field studies were performed. Clearly, it is necessary to study the production, release, transformation, and activity of the identified phenolic acids from the *P. schrenkiana* rhizophere under natural settings.

## 6. Conclusions

Rapid and radical progress of analysis technology in recent years has made it possible to isolate even minute amounts of phenolic allelochemicals and to perform sophisticated structural analyses. In addition, a wide variety of allelopathy mechanisms have been well characterised. Extensive research has been done in the field of allelopathy and, consequently, a great number of phemolic allelochemicals have been reported. The essential role that phenolic compounds play in complex interactions among living organisms in the environment is gradually unravelling. Studies of examing mixtures of phenolic compounds have shown that individual components can be additive when being evaluated for phytotoxic affects. However, further research is still needed to find evidence for the synergistic activities of phenolic acids mixtures under field conditions.

To understand the allelopathic mechanisms of phenolics more clearly, further studies on the production, role, and fate of phenolics in the ecosystem are necessary. Progress in this field will eventually lead to the development of biorational pest or weed control using allelochemicals.

## References

[1] Rice E.L. (1984). Allelopathy.

[2] Weston L.A., Duke S.O. (2003). Weed and crop allelopathy. Plant Sci..

[3] Zeng R.S., Mallik A.U., Luo S.M. (2008). Allelopathy in Sustainable Agriculture and Forestry.

[4] Molisch H. (1937). Der Einfluss einer Pflanze auf die andere-Allelopathie.

[5] Singh H.P., Kohli R.K., Batish D.R. (2001). Allelopathy in agroecosystems: An overview. J. Crop Prod..

[6] Callaway R.M., Aschehoug E.T. (2000). Invasive plants versus their new and old neighbors: A mechanism for exotic plant invasion. Science.

[7] Ridenour W.M., Callaway R.M. (2001). The relative importance of allelopathy in interference: the effects of an invasive weed on a native bunchgrass. Oecologia.

[8] Haribal M., Enwick J.A.A. (1998). Isovitexin 6″″-O-β-D-glucopyranoside: a feeding deterrent to Pieris napi oleracea from *Alliaria petiolata*. Phytochemistry.

[9] Wang Q., Ruan X., Li Z.H., Pan C.D. (2006). Autotoxicity of plants and research of coniferous forest autotoxicity. Sci. Sil. Sin..

[10] Capasso R. (1997). The chemistry, biotechnology and ecotoxicology of the polyphenols naturally occurring in vegetable wastes. Curr. Topics Phytochem..

[11] Capasso R., Cristinzio G., Evidente A., Scognamiglio F. (1992). Isolation, spectroscopy and selective phytotoxic effects of polyphenols from vegetable waste waters. Phytochemistry.

[12] Hartmann T. (1996). Diversity and variability of plant secondary metabolism: A mechanistic view. Entomol. Gen. Appl..

[13] Franz H. (2002). Secondary Metabolites as Plant Traits: Current Assessment and Future Perspectives. Plant Sci..

[14] Santana C.M., Ferrera Z.S., Padrón M.E.T., Rodríguez J.J.S. (2009). Methodologies for the extraction of phenolic compounds from environmental samples: New Approaches. Molecules.

[15] Chou C.H., Leu L.L. (1992). Allelopathic substances and interactions of *Delonix regia* (BOJ) RAF. J. Chem. Ecol..

[16] Chou C.H., Lin H.J. (1976). Autointoxication mechanism of *Oryza sativ* I. Phytotoxic effects of decomposing rice residues in soil. J. Chem. Ecol..

[17] Chou C.H., Muller C.H. (1972). Allelopathic mechanisms of *Arctostaphylos glandulosa* var. *zacaensis*. Am. Midl. Nat..

[18] Chou C.H., Lee Y.Y. (1991). Allelopathic dominance of *Miscanthus transmorrisonensis* in an alpine grassland community in Taiwan. J. Chem. Ecol..

[19] Tang C.S., Putnam A.R., Tang C.S. (1986). Continuous trapping techniques for the study of allelochemicals from higher plants. The Science of Allelopathy.

[20] Waller G.R. (1987). Atlelochemicals: Role in Agriculture and Forestry. ACS Symposium Series 330.

[21] Batish D.R., Kaur S., Singh H.P., Kohli R.K. (2008). Role of root-mediated interactions in phytotoxic interference of *Ageratum conyzoides* with rice (*Oryza sativa*). Flora.

[22] Mahall B.E., Callaway R.M. (1992). Root communication mechanisms and intercommunity distributions of 2 mojave desert shrubs. Ecology.

[23] Ghafar A., Saleem B., Qureshi M.J. (2000). Allelopathic effects of sunflower on germination and seedling growth of wheat. Pak. Pak. J. Biol. Sci..

[24] Sasikumar K., Vijayalakshmi C., Parthiban K.T. (2002). Allelopathic effects of Eucalyptus on blackgram (*Phaseolus mungo L.*). Allelopathy J..

[25] Willis R.J. (1985). The historical bases of the concept of allelopathy. J. Hist. Biol..

[26] Hostettmann K., Marston A., Hostettmann M. (1997). Preparative Chromatography Techniques: Application in Natural Product Isolation.

[27] Djurdjevic L., Mitrovic M., Pavlovic P. (2007). Methodology of allelopathy research: 2. Forest ecosystems. Allelopathy J..

[28] Putnam A.R., Tang C.S. (1986). The science of Allelopathy.

[29] Schmidt S.K. (1990). Ecological implications of the destruction of juglone (5-hydroxy-1,4-naphthoquinone) by soil bacteria. J. Chem. Ecol..

[30] Wu H., Haig T., Pratley J., Lemerle D., An M. (2001). Allelochemicals in wheat (*Triticum aestivum* L.): Production and exudation of 2,4-dihydroxy-7-methoxy-1,4- benzoxazin-3-one. J. Chem. Ecol..

[31] Inderjit (1996). Plant phenolics in allelopathy. Bot. Rev..

[32] Hadacek F. (2002). Secondary metabolites as plant traits: Current assessment and future perspectives. Crit. Rev. Plant Sci..

[33] Liebler D.C., Burr J.A., Philips L., Ham A.J.L. (1996). Gas chromatography-mass spectrometry analysis of vitamin E and its oxidation products. Anal. Biochem..

[34] Durant A.A., Fente C.A., Franco C.M., Vazquez B.I., Cepeda A. (2002). Gas-chromatography-tandem mass spectrometry determination of 17 α-ethinylestradiol residue in the hair of cattle, application to treated animals. J. Agric. Food Chem..

[35] Van Pelt C.D., Haggarty H., Brenna J.T. (1998). Quantitative subfemtomole analysis of α-tocopherol and deuterated isotopomers in plasma using tabletop GC/MS/MS. Anal. Chem..

[36] Seal A.N., Pratley J.E., Haig T, An M. (2004). Identification and quantitation of compounds in a series of allelopathic and non-allelopathic rice root exudates. J. Chem. Ecol..

[37] Politycka B. (1997). Free and glucosylated phenolics, phenol-beta-glucosyltransferase activity and membrane perability in cucumber roots affected by derivatives of cinnamic and benzoic acid. Acta Physiol. Plantarum..

[38] Cruz O.R., Anaya A.L., Hernandez-Bautista B.E. (1998). Effects of allelochemical stress produced by sicyosdeppei on seedling root ultrastructure of Phaseolous valgaris and Cucubita ficifolia. J. Chem. Ecol..

[39] Li H.H., Inoue M., Nishimura H, Mizutani J., Tsuzuki E. (1993). Interaction of trans-cinnamic acid, its related phenolic allelochemicals, and abscisic-acid in seedling growth and seed-germination of lettuce. J. Chem. Ecol..

[40] Patterson D.T. (1981). Effects of allelopathic chemicals on growth and physiological response of soybean(G*lycine max*). Weed Sci..

[41] Yu J.Q., Ye S.F., Zhang M.F., Hu W.H. (2003). Effects of root exudates and aqueous root extracts of cucumber (*Cucumis sativus*) and allelochemicals, on photosynthesis and antioxidant enzymes in cucumber. Biochem. Syst. Ecol..

[42] Rice E.L. (1974). Allelopathy.

[43] Politycka B. (1998). Phenolics and the activities of phenylalanine ammonia-1yase, phenol-beta-glucosyltransferase and beta-glucosidase in cucumber roots as affected by phenolic allelochemicals. Acta Physiol. Plantarum..

[44] Batish D.R., Singh H.P., Kaur S., Kohli R.K., Yadav S.S. (2008). Caffeic acid affects early growth, and morphogenetic response of hypocotyl cuttings of mung bean (*Phaseolus aureus*). J. Plant Physiol..

[45] Devi S.R. (1992). Effects of ferulic acid on growth and hydrolytic enzyme activities of germinating maize seeds. J. Chem. Ecol..

[46] He H.Q., Lin W.X. (2001). Studies on allelopathic physiobiochemical characteristics of rice. Chin. J. Eco-Agric..

[47] Zeng R.S., Luo S.M., Shi Y.H. (2001). Physiological and biochemical mechanism of allelopathy of secalonic acid on higher plants. Agron. J..

[48] Leslie C.A. Romani R.J. (1998). Inhibition of ethylene biosynthesis by salicylic acid. Plant Physiol..

[49] Ni H.W., Kim K.U., Shin D.H. (2000). Present status and prospect of crop allelopathy in China. Rice Allelopathy.

[50] Einhellig F.A., Galindo J.C.G., Molinillo J.M.G., Cutler H.G. (2004). Mode of allelochemical action of phenolic compounds. Allelopathy: Chemistry and Mode of Action of Allelochemicals.

[51] Watson A.K., Renney A.J. (1974). The biology of Canadian weeds. 6. *Centaurea diffusa* and *Centaurea maculosa*. Can. J. Plant Sci..

[52] Tyser R.W., Key C.H. (1988). Spotted knapweed in natural area fescue grasslands: An ecological assessment. Northwest Sci..

[53] Callaway R.M., Ridenour W.M. (2004). Novel weapons: Invasive success and the evolution of increased competitive ability. Front. Ecol. Environ..

[54] Bais H.P., Walker T.S., Stermitz F.R., Hufbauer R.A., Vivanco J.M. (2002). Enantiomeric-dependent phytotoxic and antimicrobial activity of (±)-catechin. A rhizosecreted racemic mixture from spotted knapweed. Plant Physiol..

[55] Bais H.P., Vepachedu R., Gilroy S., Callaway R.M., Vivanco J M. (2003). Allelopathy and exotic plant invasion: From molecules and genes to species interactions. Science.

[56] Perry L.G., Thelen G.C., Ridenour W.M., Weir T.L., Callaway R.M., Paschke M.W., Vivanco J.M. (2005). Dual role for an allelochemical (±)-catechin from *Centaurea maculosa* root exudates regulates conspecific seedling establishment. J. Ecol..

[57] Thelen G.C., Vivanco J.M., Newingham B., Good W., Bais H.P., Landers P., Caesar A., Callaway R.M. (2005). Insect herbivory stimulates allelopathic exudation by an invasive plant and the suppression of natives. Ecol. Lett..

[58] Weir T.L., Bais H.P., Stull V.J., Callaway R.M., Thelen G.C., Ridenour W.M., Bhamidi S., Stermitz F. R., Vivanco J. M. (2006). Oxalate contributes to the resistance of *Gaillardia grandiflora* and *Lupinus sericeus* to a phytotoxin produced by *Centaurea maculosa*. Planta.

[59] Weir T.L., Bais H.P., Vivanco J.M. (2003). Intraspecific and interspecific interactions mediated by a phytotoxin, (−)-catechin, secreted by the roots of *Centaurea maculosa* (spotted knapweed). J. Chem. Ecol..

[60] Perry L.G., Johnson C., Alford É.R., Vivanco J.M., Paschke M.W. (2005). Screening of grassland plants for restoration after spotted knapweed invasion. Restor. Ecol..

[61] Thorpe A. (2006). Biochemical effects of Centaurea maculosa on soil nutrient cycles and plant communities.

[62] Blair A.C., Hanson B.D., Brunk G.R., Marrs R.A., Westra P., Nissen S.J., Hufbauer R.A. (2005). New techniques and findings in the study of a candidate allelochemical implicated in invasion success. Ecol. Lett..

[63] Blair A.C., Nissen S.J., Brunk G.R., Hufbauer R.A. (2006). A lack of evidence for an ecological role of the putative allelochemical (±)-catechin in *Centaurea maculosa* invasion process. J. Chem. Ecol..

[64] Duke S.O., Blaie A.C., Dayan F.E., Johnson R.D., Meepagala K.M., Cook D., Bajsa J. (2009). Is (-)-cathchin a novel weapon of Spooted Knapweed (*Centaurea stoebe*)?. J. Chem. Ecol..

[65] Tharayil N., Bhowmik P.C., Xing B. (2008). Bioavailability of allelochemicals as affected by companion compounds in oil matrices. J. Agric. Food Chem..

[66] Blair A.C., Weston L.A., Nissen S.J., Brunk G.R., Hufbauer R. (2009). The importance of analytical techniques in allelopathy studies with the reported allelochemical catechin as an example. Biol. Invasions.

[67] Furubayashi A., Hiradate S., Fujii Y. (2007). Role of catechol structure in the adsorption and transformation reactions of L-DOPA in soils. J. Chem. Ecol..

[68] Inderjit, Pollock J., Callaway R.M., Hoben W. (2008). Phytotoxic effects of (±)-catechin *in vitro*, in soil, and in the field. Plos One.

[69] Vivanco J.M., Bais H.P., Stermitz F.R., Thelen G.C., Callaway R.M. (2004). Biogeographical variation in community response to root allelochemistry: Novel weapons and exotic invasion. Ecol. Lett..

[70] Norton A.P., Blair A.C., Hardin J.G., Nissen S.J., Brunk G.R. (2008). Herbivory and novel weapons: No evidence for enhanced competitive ability or allelopathy induction of *Centaurea diffusa* by biological controls. Biol. Invasions.

[71] Romagni J.G., Allen S.N., Dayan F.E. (2000). Allelopathic effects of volatile cineoles on two weedy plant species. J. Chem. Ecol..

[72] Duke S.O., Dayan F.E., Hernández A., Duke M.V., Abbas H.K. (1997). Natural products as leads for new herbicide modes of action. In Proceedings Brighton Crop Protection Conference---Weeds, Brighton, UK.

[73] Rimando A.M., Olofsdotter M., Dayan F.E., Duke S.O. (1999). Searching for Rice Allelochemicals. Agron. J..

[74] Nakano H., Fujii Y., Suzuki T., Yamada K., Kosemura S.S., Yamamura S., Suzuki T., Hasegawa K. (2001). A growth-inhibitory substance exuded from freeze-dried mesquite (*Prosopis juliﬂora* (Sw.) DC.) leaves. Plant Growth Regul..

[75] Iqbal Z., Hiradate S., Noda A., Fujii Y. (2003). Allelopathic activity of buckwheat: Isolzation and characterization of phenolics. Weed Sci..

[76] Shiraishi S., Watanabe I., Kuno K., Fujii Y. (2005). Evaluation of the allelopathic activity of ﬁve Oxalidaceae cover plants and the demonstration of potent weed suppression by Oxalis species. Weed Biol. Manag..

[77] Parvez M.M., Tomita-Yokotani K., Fujii Y., Konishi T., Iwashina T. (2004). Effects of quercetin and its seven derivatives on the growth of *Arabidopsis thaliana* and *Neurospora crassa*. Biochem. Syst. Ecol..

[78] Inderjit, Callaway R.M. (2003). Experimantal designs for the study of allelopathy. Plant Soil..

[79] Inderjit, Kaur M., Foy C.L. (2001). On the significance of field studies in allelopathy. Weed Technol..

[80] Ai Hamdi B., Inderjit, Olofsdotter M., Streibig J.C. (2001). Laboratory bioassay for phytotoxicity: An example from wheat straw. Agron J..

[81] Yu J.Q., Matsui Y. (1997). Effects of root exudates of cucumber (*Cucumis sativus*.) and allelochemicals on ion uptake by cucumber seedlings. J. Chem. Ecol..

[82] Yu J.Q., Matsui Y. (1994). Phytotoxic substances in root exudates of cucumber (*Cucumis sativus* L.). J. Chem. Ecol..

[83] Ye S.F., Zhou Y.H., Sun Y., Zou L.Y., Yu J.Q. (2006). Cinnamic acid causes oxidative stress in cucumber roots, and promotes incidence of *Fusarium* wilt. Environ. Exp. Bot..

[84] Luo X., Pan C.D., Huang M.M., Liu C.L. (2006). Autotoxicity of *Picea schrenkiana* litter aqueous extracts on seed germination and seedling growth. Xinjiang Agr. Sci..

[85] Wang Q., Ruan X., Pan C.D;, Xu N.Y., Luo X., Huang M.M. (2006). Need for sustainability policy—A case study of the National Forest Conservation Program (NFCP) in the western region of Tianshan Mountain, China. Forest. Chron..

[86] Appel H.M. (1993). Phenolics in ecological interactions: The importance of oxidation. J. Chem. Ecol..

[87] Blum U. (1996). Allelopathic interactions involving phenolic acids. J. Nematol..

[88] Blum U., Dalton B.R. (1985). Effects of ferulic acid, an allelopathic compound on leaf expansion of cucumber seedlings in nutrient culture. J. Chem. Ecol..

[89] Blum U., Shafer S.R. (1988). Microbial populations and phenolic acids in soil. Soil Biol. Biochem..

[90] Blum U. (1998). Effects of microbial utilization of phenolic acids and their phenolic acid breakdown products on allelopathic interactions. J. Chem. Ecol..

